# Regulation of vitamin D system in skeletal muscle and resident myogenic stem cell during development, maturation, and ageing

**DOI:** 10.1038/s41598-020-65067-0

**Published:** 2020-05-19

**Authors:** Ratchakrit Srikuea, Muthita Hirunsai, Narattaphol Charoenphandhu

**Affiliations:** 1 Department of Physiology, Faculty of Science, Mahidol University, Bangkok, 10400 Thailand; 2 Department of Biopharmacy, Faculty of Pharmacy, Srinakharinwirot University, Nakhon Nayok, 26120 Thailand; 3 Center of Calcium and Bone Research (COCAB), Faculty of Science, Mahidol University, Bangkok, 10400 Thailand; 4 Institute of Molecular Biosciences, Mahidol University, Nakhon Pathom, 73170 Thailand; 5 The Academy of Science, The Royal Society of Thailand, Dusit, Bangkok, 10300 Thailand

**Keywords:** Ageing, Calcium and vitamin D

## Abstract

Skeletal muscle exhibits enormous plasticity throughout life, however, less is known regarding how the stages of growth regulate its local vitamin D system. Herein, we investigated serum 25(OH)D_3_ and Ca^2+^ levels along with the vitamin D system in skeletal muscle and resident myogenic stem cells of male C57BL/6 mice during development, maturation, and ageing. Compared with development, significant increases in vitamin D receptor (VDR) protein expression in mature and aged muscles were associated with increased serum 25(OH)D_3_ and centronucleated fibres, respectively. The substantial increase in VDR protein expression in aged muscle was also related to reduced downstream mTOR signalling protein expression which was more pronounced in fast-glycolytic compared to slow-oxidative muscles. Intriguingly, serum Ca^2+^ and vitamin D-metabolising enzyme (CYP27B1 and CYP24A1) levels in skeletal muscle were not different across age. In primary cell culture, nuclear VDR protein was expressed in undifferentiated skeletal muscle stem cells (SMSC) after 1α,25(OH)_2_D_3_ treatment. Additionally, a diminished response to 1α,25(OH)_2_D_3_ was observed with age as there was a rapid commitment of SMSC towards differentiation under growth-stimulating conditions. Collectively, understanding the local vitamin D system in skeletal muscle could help develop effective interventions for vitamin D supplementation to improve skeletal muscle mass and function during ageing.

## Introduction

Vitamin D research has primary focused on the role of Ca^2+^ homeostasis regulation in classical vitamin D target tissues, i.e., bone, intestine, and kidney^1,2^. However, non-calcemic action of vitamin D has been suggested as expression of vitamin D receptor (VDR) and vitamin D-metabolising enzymes (CYP27B1 and CYP24A1) has been reported in non-classical vitamin D target tissues, i.e., brain and heart^3^ and skeletal muscle^4^. This emerging evidence has suggested possible novel functions of vitamin D besides calcium homeostasis as well as how the vitamin D system is regulated in these non-classical vitamin D target tissues.

In skeletal muscle, VDR protein has been reported to be expressed in both skeletal muscle cells and tissue, including mouse skeletal muscle cell line (C2C12)^4–9^, primary skeletal muscle cells^10,11^, human skeletal muscle cells^12–14^, rodent skeletal muscle^4,6,9,10,15–18^, and human skeletal muscle^12,14,19–21^. In addition, vitamin D system-related proteins (VDR, CYP27B1, and CYP24A1) are expressed in regenerating muscle along with expression of VDR in skeletal muscle stem cells (SMSC) after injury^4^, suggesting a contribution of this resident myogenic stem cell (namely satellite cell) in the local vitamin D system during skeletal muscle regeneration. However, less is known regarding the regulation of the vitamin D system in skeletal muscle in a fibre-type specific manner. Data in the predominantly fast-twitch tibialis anterior revealed alterations in vitamin D system regulation during recovery from injury^4^.

Skeletal muscle consists of slow-oxidative (type I) and fast-glycolytic (type II) muscle fibres, which are characterized based on the abundant of slow and fast myosin heavy chain (MHC) expression in rodents and humans^22^. These characteristics of muscle fibre type directly affect skeletal muscle function, i.e., force production, speed of contraction, and fatigue resistance^23^. Providing clinical relevance, the vitamin D_3_ deficiency (serum 25(OH)D_3_ < 20 ng/ml)^24^ has been associated with muscle weakness in older adults^25,26^. Therefore, vitamin D_3_ supplementation has been recommended to older adults who have a vitamin D deficiency in order to improve skeletal muscle function^27–30^. However, whether regulation of the vitamin D system in skeletal muscle is differentially regulated among fibre types during ageing is currently unknown.

Additionally, specific expression of the VDR protein in SMSC during muscle regeneration has been demonstrated^4^. This finding suggests a potential connection of non-calcemic action of vitamin D on the regulation of SMSC activity. Skeletal muscle is a post-mitotic tissue and it relies on SMSC function^31^. During development, rapid skeletal muscle growth also represents an active stage for SMSC^32^. In contrast, SMSC becomes quiescent during maturation but can be activated upon muscle injury^33^. Ageing reduces the proliferative potential of SMSC which can decrease the regenerative capacity of aged muscle^34^. However, the effect of age on vitamin D regulation of SMSC function at distinct stages of muscle growth is an unresolved question. Previously, the active form of vitamin D_3_ [1α,25(OH)_2_D_3_] has been shown to regulate the expression of VDR and vitamin D-metabolising enzymes (CYP27B1 and CYP24A1) in primary muscle cells^10^. A limitation of that study was that, the primary muscle cells were isolated from 3 week-old developing muscle. Hence, it is difficult to extrapolate these findings to mature and aged SMSCs.

Therefore, the purpose of this study was to investigate the local vitamin D system in slow-oxidative (type I) and fast-glycolytic (type II) muscles and its associations with skeletal muscle plasticity during development, maturation, and ageing. Additionally, we examined the expression of VDR and vitamin D-metabolising enzymes (CYP27B1 and CYP24A1) in SMSCs isolated from developmental, mature, and aged muscles to reveal how age affects the SMSC response to vitamin D_3_.

## Results

### Fibre type composition in slow-oxidative and fast-glycolytic muscles during development, maturation, and ageing

**Figure 1:**
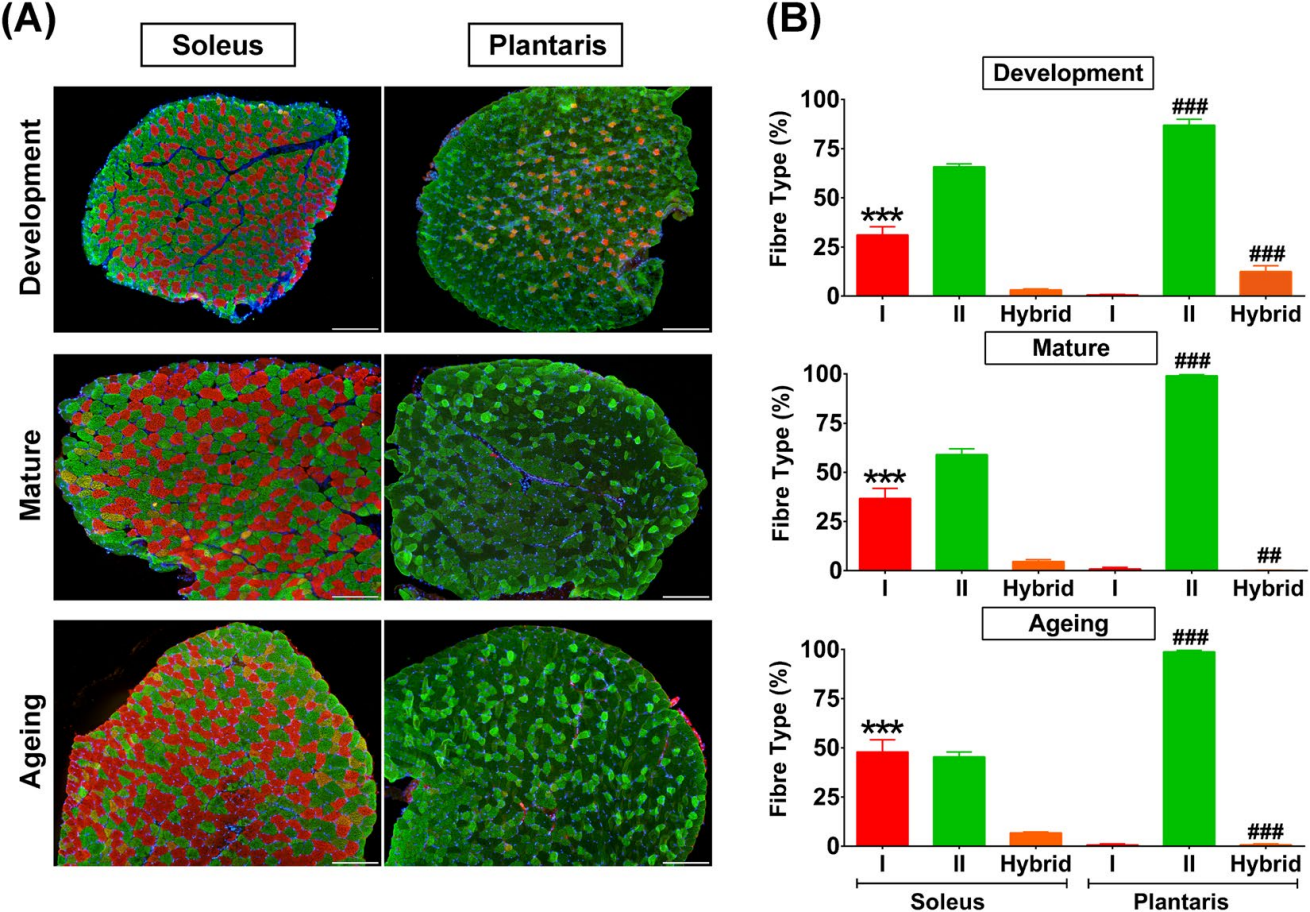
Fibre type composition in developmental, mature, and aged soleus and plantaris muscles. (**A**) Representative images of fibre type composition in soleus and plantaris muscles at different stages of growth. Type I fibres (red), type II fibres (green), and hybrid fibres (orange). Images were taken at ×100 magnification, scale bars = 200 µm. (**B**) Quantitative analysis of fibre type composition during development, maturation, and ageing (n = 6 mice/group). ***p < 0.0001 compared to type I fibre of plantaris muscle, ^##^*p* < 0.001 and ^###^*p* < 0.0001 compared to hybrid fibre/type II fibre of soleus muscle.

To assess the potential contribution of muscle fibre type on the regulation of the vitamin D system, we studied the slow-oxidative (soleus) and fast-glycolytic (plantaris) muscles at different stages of growth as illustrated in Fig. 1A. The ageing group in this study represents an early stage of ageing to control for age-related comorbid conditions that could confound the interpretation of the results on vitamin D system in skeletal muscle. The quantitative analysis revealed a remarkable significant difference of type I muscle fibre distribution, which was higher in the soleus than plantaris muscle at development (31.12 ± 1.74% vs. 0.65 ± 0.11%, *p* < 0.0001), maturation (36.64 ± 2.08% vs. 0.91 ± 0.31%, *p* < 0.0001), and ageing (47.89 ± 2.48% vs. 0.58 ± 0.20%, *p* < 0.0001) (Fig. 1B). In contrast, type II muscle fibre frequency was significantly higher in plantaris than soleus muscle in development (86.90 ± 1.24% vs. 65.75 ± 1.52%, *p* < 0.0001), maturation (99.02 ± 0.31% vs. 58.95 ± 3.07%, *p* < 0.0001), and ageing (98.76 ± 0.29% vs. 45.38 ± 2.47%, *p* < 0.0001) (Fig. 1B). Nevertheless, hybrid muscle fibre frequency (co-expressions of fast MHC and slow MHC isoforms) was significantly higher in the plantaris compared to soleus muscle during development (12.45 ± 1.21% vs. 3.14 ± 0.59%, *p* < 0.0001). This difference was reversed during advanced age where the plantaris muscle had a significantly lower proportion of hybrid fibres than the soleus muscle during maturation (0.10 ± 0.05% vs. 4.67 ± 0.94%, *p* < 0.001) and ageing (0.66 ± 0.21% vs. 6.73 ± 0.53%, *p* < 0.0001) (Fig. 1B). Altogether, these difference show distinct skeletal muscle properties between soleus and plantaris muscles at different stages of growth.

### Characteristics of slow-oxidative and fast-glycolytic muscles at different stages of growth

**Figure 2:**
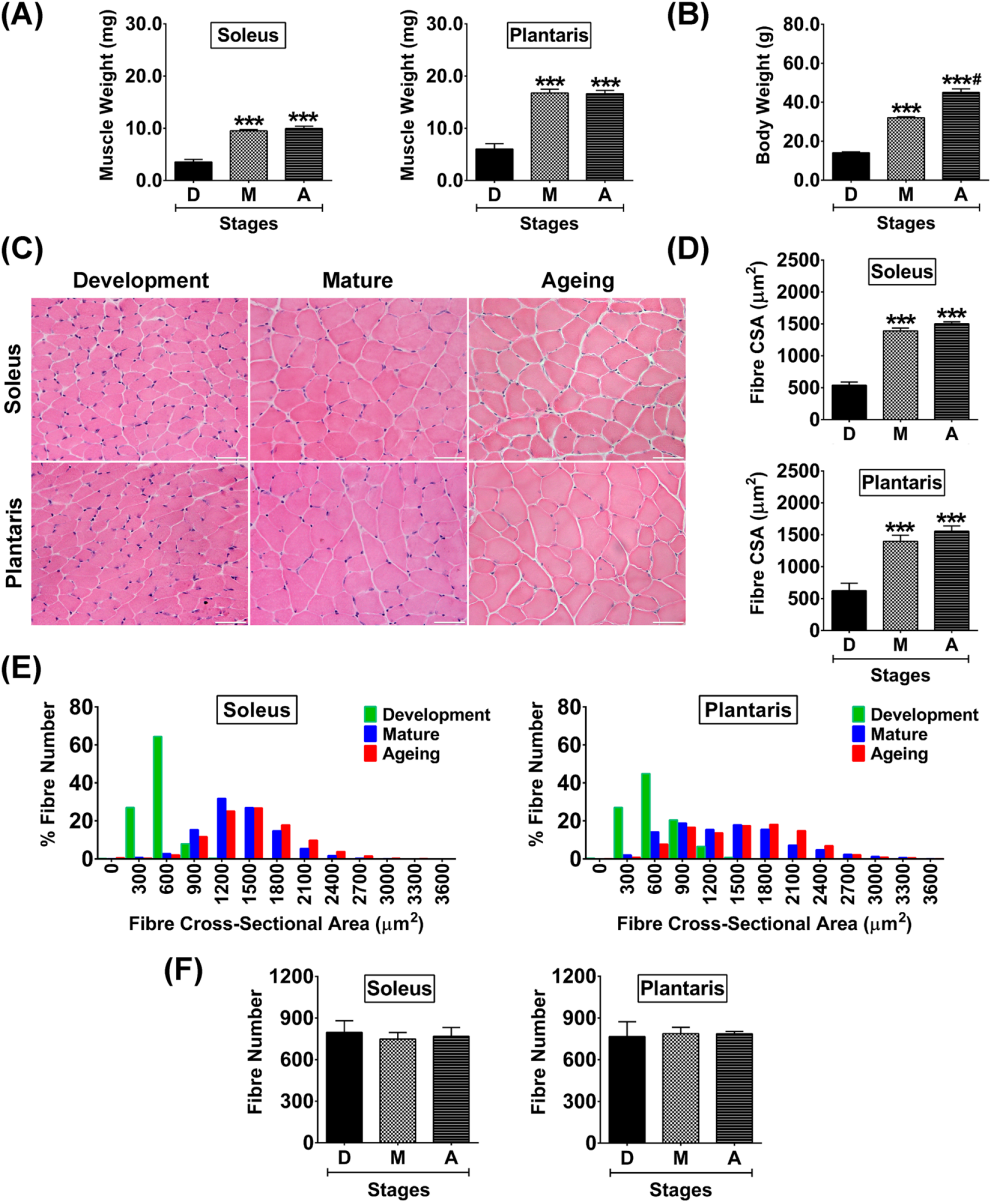
Muscle weight, body weight, and histological characteristics of soleus and plantaris muscles during development, maturation, and ageing. (**A**) Muscle weight, (**B**) Body weight, (**C**) Representative hematoxylin and eosin-stained images of developmental, mature, and aged muscles, (**D)** Quantitative analysis of fibre cross-sectional area (CSA), (**E**) Histogram analysis of muscle fibre size distribution, and (**F**) Quantitative analysis of fibre number. Images were taken at ×400 magnification, scale bars = 50 µm. ***p < 0.001 compared to developmental stage, ^#^*p* < 0.001 compared to maturation stage (n = 6 mice/group).

Although the different in fibre type composition in slow-oxidative (soleus) and fast-glycolytic (plantaris) muscles was demonstrated, skeletal muscle plasticity during the different stages of growth are comparable. Muscle wet weight was significantly increased in parallel with body weight in soleus (*p* < 0.001) and plantaris (*p* < 0.001) muscles during development to maturation (Fig. 2A). There was no further increase in muscle wet weight during maturation to ageing (Fig. 2A), despite a progressive increase in body weight (Fig. 2B). To support findings in tissue wet weight, histological analysis across ages (Fig. 2C) revealed muscle fibre CSA was significantly increased during maturation (*p* < 0.001) and ageing (*p* < 0.001) compared to development in both muscles (Fig. 2D). However, fibre size, as demonstrated by histogram analysis showed differences between soleus and plantaris muscles during maturation and ageing (Fig. 2E). The frequency distribution of soleus muscle fibre size was concentrated at a median level. This distribution pattern differs from the plantaris, which showed a more widespread fibre size distribution, suggesting higher variation of muscle fibre size. In contrast to the changes of fibre size, the number of fibres in the soleus and plantaris were not significantly different at any age investigated (Fig. 2F).

### Serum levels of 25(OH)D_3_/D_2_, 3-Epi-25(OH)D_3_/D_2_, and total Ca^2+^ during advanced age

**Figure 3:**
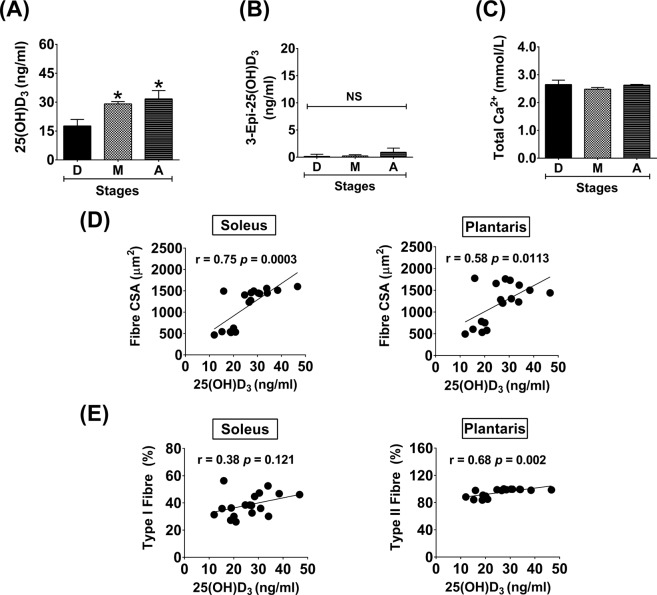
Serum levels of 25(OH)D_3_, 3-Epi-25(OH)D_3_, and total Ca^2+^ during advanced age and the association of 25(OH)D_3_ level and skeletal muscle plasticity. (**A**) 25(OH)D_3_, (**B**) 3-Epi-25(OH)D_3_, (**C**) Total Ca^2+^, (**D**) Correlation analysis between 25(OH)D_3_ level and fibre CSA, and (**E**) Correlation analysis between 25(OH)D_3_ level and fibre type. *p < 0.05 compared to developmental stage (n = 6 mice/group). NS indicates not statistically significant.

In this study, mice were fed with a standard diet containing 205 IU/100 g vitamin D_3_ throughout the study period, however, serum 25(OH)D_3_ level (17.7 ± 1.4 ng/ml) during development was lower than the normal range for vitamin D status (∼30 ng/ml). Serum 25(OH)D_3_ level was significantly greater during maturation (29.1 ± 1.2 ng/ml) (*p* < 0.05) and ageing (31.7 ± 4.4 ng/ml) (*p* < 0.05) compared to development (Fig. 3A). In contrast to 25(OH)D_3_, 3-Epi-25(OH)D_3_ was barely detected in serum samples and no significant differences between growth stages was ascertained (Fig. 3B). Neither 25(OH)D_2_ nor 3-Epi-25(OH)D_2_ were detected in any samples. Additionally, there was no association between serum 25(OH)D_3_ and total Ca^2+^ level at different stages of growth (Fig. 3C).

### Associations of serum 25(OH)D_3_ level and skeletal muscle plasticity

According to the changes of serum 25(OH)D_3_ during different stages of growth, 25(OH)D_3_ level had a positive relationship with slow-oxidative (soleus) muscle fibre CSA (r = 0.75 *p* = 0.0003) and fast-glycolytic (plantaris) muscle fibre CSA (r = 0.58 *p* = 0.0113) (Fig. 3D). Moreover, serum 25(OH)D_3_ level positively correlated with the percentage of type II fibres in plantaris muscle (r = 0.68 *p* = 0.002) but not type I fibres of soleus muscle (r = 0.38 *p* = 0.121) (Fig. 3E).

### Vitamin D system in slow-oxidative and fast-glycolytic muscles during advanced age

**Figure 4:**
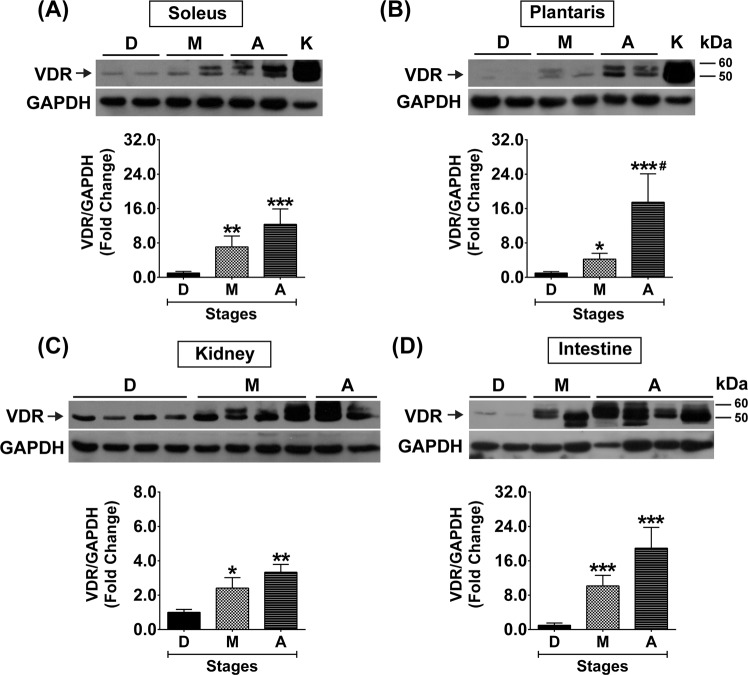
VDR protein expression in soleus and plantaris muscles, kidney, and intestine during development, maturation, and ageing. VDR protein expression (**A**) soleus muscle and (**B**) plantaris muscle (n = 6 mice/group). K = Kidney samples serve as a positive control for VDR protein expression in soleus and plantaris muscles. (**C**,**D**) VDR protein expression in the vitamin D-sensitive tissues including (**C**) kidney (n = 6 mice/group) and (**D**) intestine (n = 5-6 mice/group). *p < 0.05, **p < 0.01, ***p < 0.001 compared to developmental stage, ^#^*p* < 0.05 compared to maturation stage. The highly sensitive VDR (D-6) antibody was used to detect VDR protein expression and GAPDH served as loading control. VDR protein expression level was normalized with GAPDH protein expression that obtained from the same gel and experiment.

**Figure 5:**
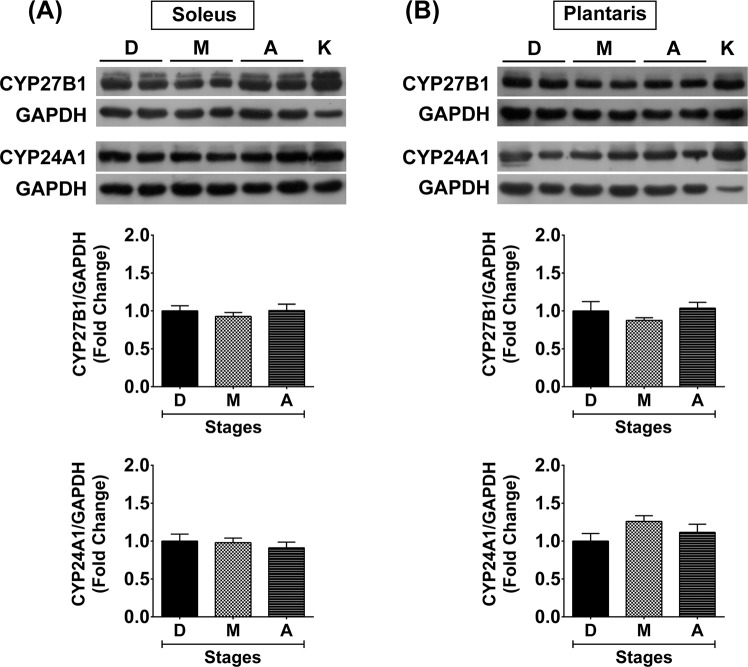
Expression of vitamin D-metabolising enzymes in soleus and plantaris muscles during development, maturation, and ageing. CYP27B1 and CYP24A1 protein expression (**A**) soleus muscle and (**B**) plantaris muscle. GAPDH served as loading control (n = 6 mice/group), K = Kidney sample (positive control). CYP27B1 and CYP24A1 protein expression level was normalized with GAPDH protein expression that obtained from the same gel and experiment.

To clarify whether skeletal muscle plasticity affects the vitamin D system in skeletal muscle, the expression of vitamin D system-related proteins (VDR, CYP27B1, and CYP24A1) in slow-oxidative (soleus) and fast-glycolytic (plantaris) muscles at different stages of growth were investigated. During development, VDR protein was expressed at low level in the soleus muscle but significantly increased during maturation (7.1 ± 2.5-fold) (*p* < 0.01) and ageing (12.3 ± 3.6-fold) (*p* < 0.001) (Fig. 4A). However, VDR protein was barely detectable in the plantaris muscle during development but significantly increased during maturation (4.2 ± 1.4-fold) (*p* < 0.05) and increased at a substantial level during ageing (17.5 ± 6.6-fold) (*p* < 0.001) (Fig. 4B). These temporal changes in VDR protein expression were coordinated with VDR protein expression in kidney and intestine which are vitamin D-sensitive tissues (Fig. 4C,D). Additionally, VDR protein expression in soleus (r = 0.68 *p* = 0.002) and plantaris (r = 0.65 *p* = 0.004) muscles positively correlated with serum 25(OH)D_3_ level. Contrary to VDR protein expression, vitamin D-metabolising enzyme (CYP27B1 and CYP24A1) protein expression was not significantly different at any age in soleus muscle (Fig. 5A) and plantaris muscle (Fig. 5B).

### Expression and localisation of vitamin D system in slow-oxidative and fast-glycolytic muscles

**Figure 6:**
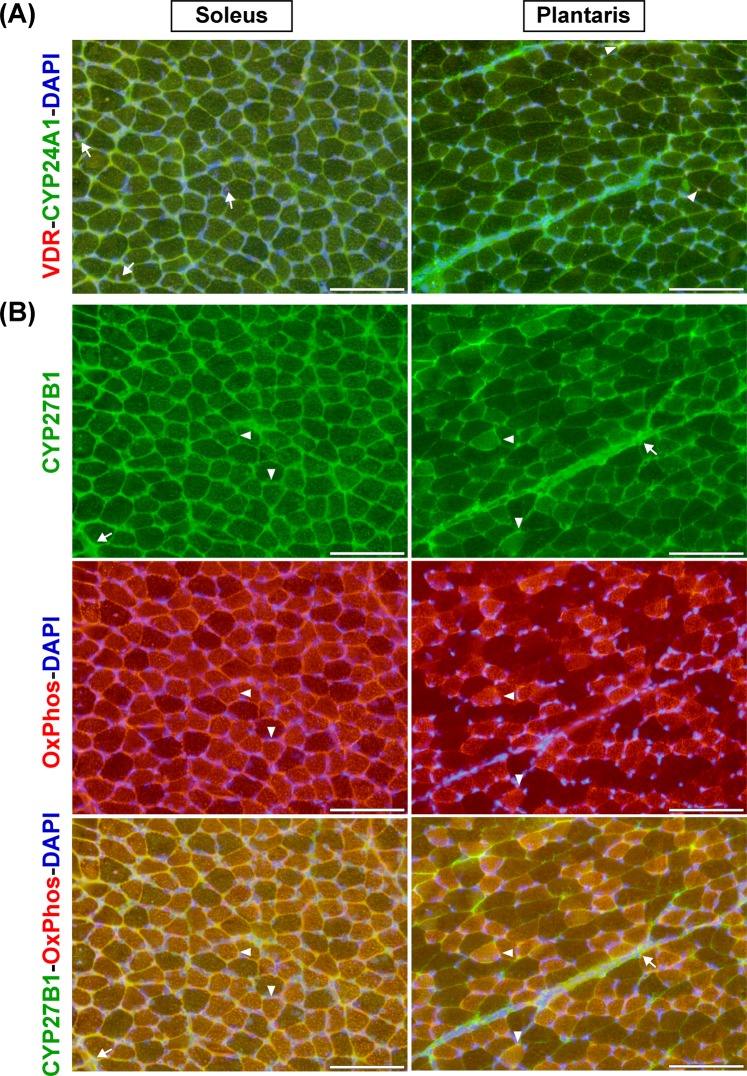
Expression and localisation of vitamin D system-related proteins in soleus and plantaris muscles. (**A**) VDR and CYP24A1 protein expression; VDR protein was expressed in centronucleated muscle fibres of soleus muscle (arrows) while co-localisation of VDR and CYP24A1 protein expression (arrowheads) was observed in plantaris muscle. (**B**) CYP27B1 and OxPhos protein expression; CYP27B1 protein mainly expressed in mitochondrial compartment (arrowheads) as indicated by OxPhos staining and expressed in extracellular matrix (arrows) in soleus and plantaris muscles. The representative images of VDR-CYP24A1-DAPI and CYP27B1-OxPhos-DAPI staining are illustrated using serial-sections. DAPI was used to delineate nuclear localisation of the stained-sections. Images were taken at ×200 magnification, scale bars = 100 µm.

The expression and localisation of vitamin D system-related proteins (VDR, CYP27B1, and CYP24A1) in slow-oxidative (soleus) and fast-glycolytic (plantaris) muscles during development that could impact vitamin D action during advanced age are illustrated in Fig. 6. VDR protein could be detected in centronucleated muscle fibres in soleus muscle (arrows). In contrast, co-localisation of VDR and CYP24A1 protein expression (arrowheads) was observed in plantaris muscle (Fig. 6A). CYP27B1 protein expression in soleus and plantaris muscles tended to localise in the mitochondrial compartment (arrowheads) as indicated by OxPhos staining and was also expressed in the extracellular matrix compartment (arrows) (Fig. 6B).

### VDR protein expression in aged muscle associated with increased centronucleated muscle fibres and decreased phosphorylation of downstream mTOR signalling proteins

**Figure 7:**
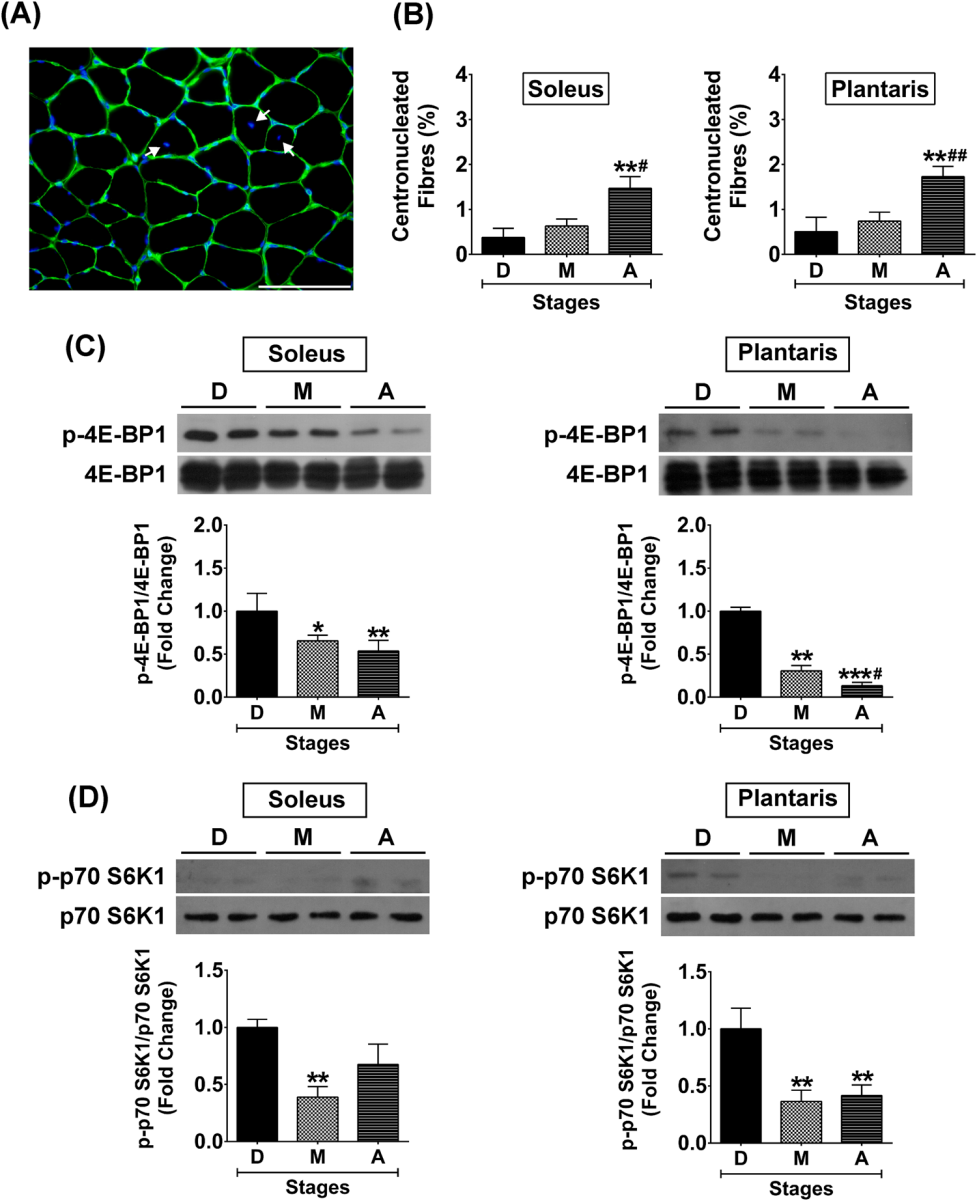
Centronucleated muscle fibres and downstream mTOR signalling protein expression in soleus and plantaris muscles during development, maturation, and ageing. (**A**) Representative image of centronucleated muscle fibres, (**B**) Percentage of centronucleated muscle fibres at different stages of growth, (**C**) Phosphorylation of 4E-BP1/4E-BP1 protein expression level, and (**D**) Phosphorylation of p70 S6K1/p70 S6K1 protein expression level. Image was taken at ×400 magnification, scale bar = 50 µm. *p < 0.05, **p < 0.01, ***p < 0.001 compared to developmental stage and ^#^*p* < 0.05, ^##^*p* < 0.01 compared to maturation stage (n = 6 mice/group).

To elucidate how VDR protein expression was substantial increased in the aged muscle, the percentage of centronucleated muscle fibres (Fig. 7A) that could be observed during ageing was evaluated. The results revealed that the percentage of centronucleated muscle fibres was significantly greater in soleus muscle (0.38 ± 0.08% vs. 1.47 ± 0.26%, *p* < 0.01) and plantaris muscle (0.51 ± 0.13% vs. 1.73 ± 0.23%, *p* < 0.01) when development and ageing were compared (Fig. 7B). These results suggest an increase in SMSC activity to maintain muscle integrity during ageing that might contribute to an increase in VDR protein expression in the aged muscle. In addition, phosphorylation of downstream mTOR signalling proteins that regulate skeletal muscle protein synthesis^35^ were different in development and ageing. Phosphorylation of 4E-BP1 protein was significantly decreased during ageing to a greater degree in the plantaris muscle (1.00 ± 0.02-fold vs. 0.13 ± 0.04-fold, *p* < 0.001) as compared to the soleus muscle (1.00 ± 0.08-fold vs. 0.54 ± 0.12-fold, *p* < 0.01) (Fig. 7C). Additionally, phosphorylation of p70 S6K1 protein was significantly decreased only in aged plantaris muscle (1.00 ± 0.07-fold vs. 0.42 ± 0.09-fold, *p* < 0.001) but not aged soleus muscle (1.00 ± 0.03-fold vs. 0.68 ± 0.18-fold, *p* > 0.05) (Fig. 7D). The significantly lower of 4E-BP1 and p70 S6K1 phosphorylation in aged plantaris muscle suggest a decrease in protein synthesis that might augment the substantial elevation of VDR protein expression compared to aged soleus muscle.

### Expression of vitamin D system-related proteins in SMSC

**Figure 8:**
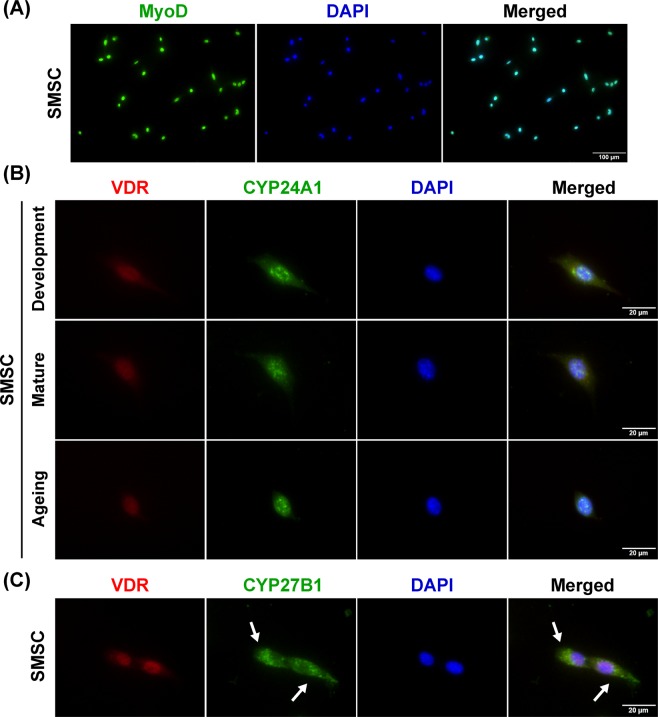
Expression and localisation of vitamin D system-related proteins in SMSC. (**A**) Representative image of purified SMSCs (MyoD^+^ cells), (**B**) Representative images of VDR and CYP24A1 protein expression in developmental, mature, and aged SMSCs. Co-localisation of VDR and CYP24A1 protein expression in the nucleus (DAPI-stained) was demonstrated in SMSCs after treated with 100 nM 1α,25(OH)_2_D_3_ for 24 h. (**C**) CYP27B1 protein expression was localised in the cytoplasmic compartment of SMSCs (arrows) in response to 100 nM 1α,25(OH)_2_D_3_-treated for 24 h. Images of A and B-C were taken at ×200 and ×400 magnifications, respectively.

To further validate a role for the vitamin D system in skeletal muscle tissue, the expression of vitamin D system-related proteins (VDR, CYP27B1, and CYP24A1) were investigated in isolated SMSCs. The purity of SMSC in this study was determined using MyoD staining to evaluate the expression of this myogenic regulatory factor in cultured SMSC (Fig. 8A). The results demonstrated that cultured SMSC (MyoD^+^ cells) derived from different growth stages expressed VDR protein in responses to 1α,25(OH)_2_D_3_ treatment (Fig. 8B). In addition, CYP24A1 protein, responsible for the catabolism of vitamin D in target cells, was expressed and co-localised with VDR protein in developmental, mature, and aged SMSCs treated with 1α,25(OH)_2_D_3_ (Fig. 8B). In contrast, SMSCs expressed CYP27B1 protein mainly in the cytoplasmic compartment (arrows) and no co-localisation with VDR protein after 1α,25(OH)_2_D_3_ treatment was observed (Fig. 8C). These results suggest that SMSC is a source of vitamin D system-related proteins in skeletal muscle and this resident myogenic stem cell could be a direct target of vitamin D_3_ during skeletal muscle plasticity changes that occur in advanced age.

### Decreased responsiveness of SMSC to 1α,25(OH)_2_D_3_ during advanced age

**Figure 9:**
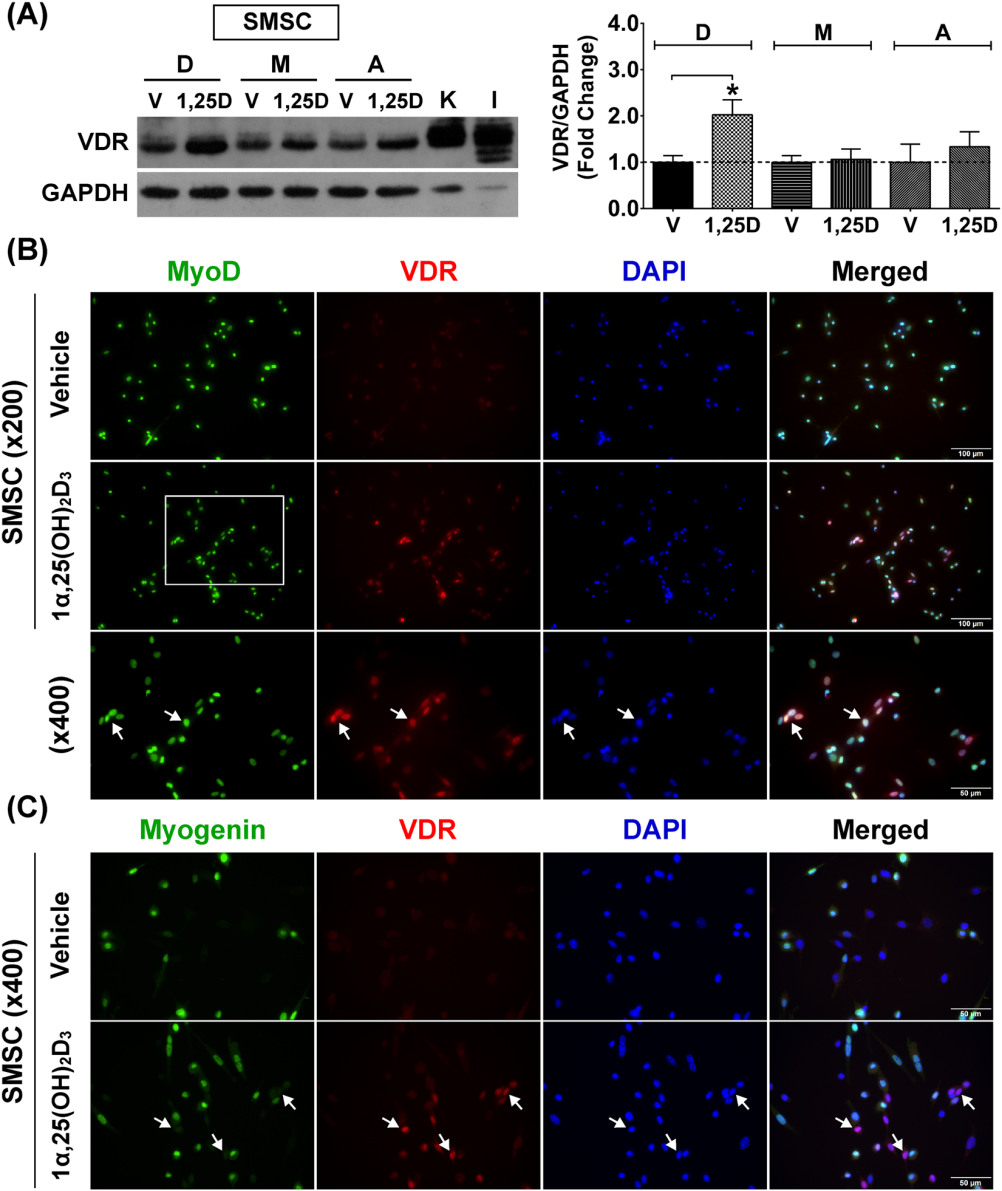
State-specific VDR protein expression of SMSCs in response to 1α,25(OH)_2_D_3_ treatment. (**A**) Representative immunoblot of VDR protein expression of developmental (**D**), mature (M), and aged (**A**) SMSCs after vehicle-treated and 100 nM 1α,25(OH)_2_D_3_-treated for 48 h (daily-treated). The highly sensitive VDR (D-6) antibody was used to detect VDR protein expression and GAPDH served as loading control. K = Kidney and I = Intestine (positive controls). VDR protein expression level was normalized with GAPDH protein expression that obtained from the same gel and experiment. Data obtained from 1α,25(OH)_2_D_3_-treated group was normalized with the respective vehicle-treated group to demonstrate the fold change at each growth stage (n = 3 SMSCs isolated from different mice/growth stage). *p < 0.05 compared to vehicle-treated group (developmental SMSC). (**B**) VDR protein expression (arrows) in developmental SMSC (MyoD^+^ cells) was substantial increased after treated with 100 nM 1α,25(OH)_2_D_3_ for 48 h (daily-treated). (**C**) VDR protein specifically expressed in undifferentiated SMSC (Myogenin^-^ cells) (arrows) after treated with 100 nM 1α,25(OH)_2_D_3_ for 48 h (daily-treated). DAPI was used to visualize nuclear localisation. Image (**B**,**C**) were taken at ×200 and ×400 magnifications. Rectangular line in (**B**) represents the area that is illustrated at ×400 magnification.

To evaluate the response of SMSC to vitamin D_3_ during advanced age, VDR protein expression in response to 1α,25(OH)_2_D_3_ treatment was examined in SMSCs derived from developmental, mature, and aged muscles (Fig. 9A). The results revealed that VDR protein expression in developmental SMSC was significantly increased after treatment with 1α,25(OH)_2_D_3_ compared to vehicle-treated control (2.0 ± 0.3-fold) (*p* < 0.05). In contrast, the responses to 1α,25(OH)_2_D_3_ treatment was diminished in mature SMSC (1.1 ± 0.1-fold) and aged SMSC (1.3 ± 0.2-fold) as compared with their respective vehicle-treated controls. To support this notion, the expression and localisation of VDR protein in developmental SMSC in response to vehicle treatment or 1α,25(OH)_2_D_3_ treatment conditions are illustrated in Fig. 9B. An increase in nuclear localisation of VDR protein expression was demonstrated after 1α,25(OH)_2_D_3_ treatment (arrows). Taken together, it appears that SMSC is a vitamin D_3_-sensitive cell, however, the response of SMSC to the active form of vitamin D_3_ declined during advanced age.

### State-specific VDR protein expression in undifferentiated SMSC

**Figure 10:**
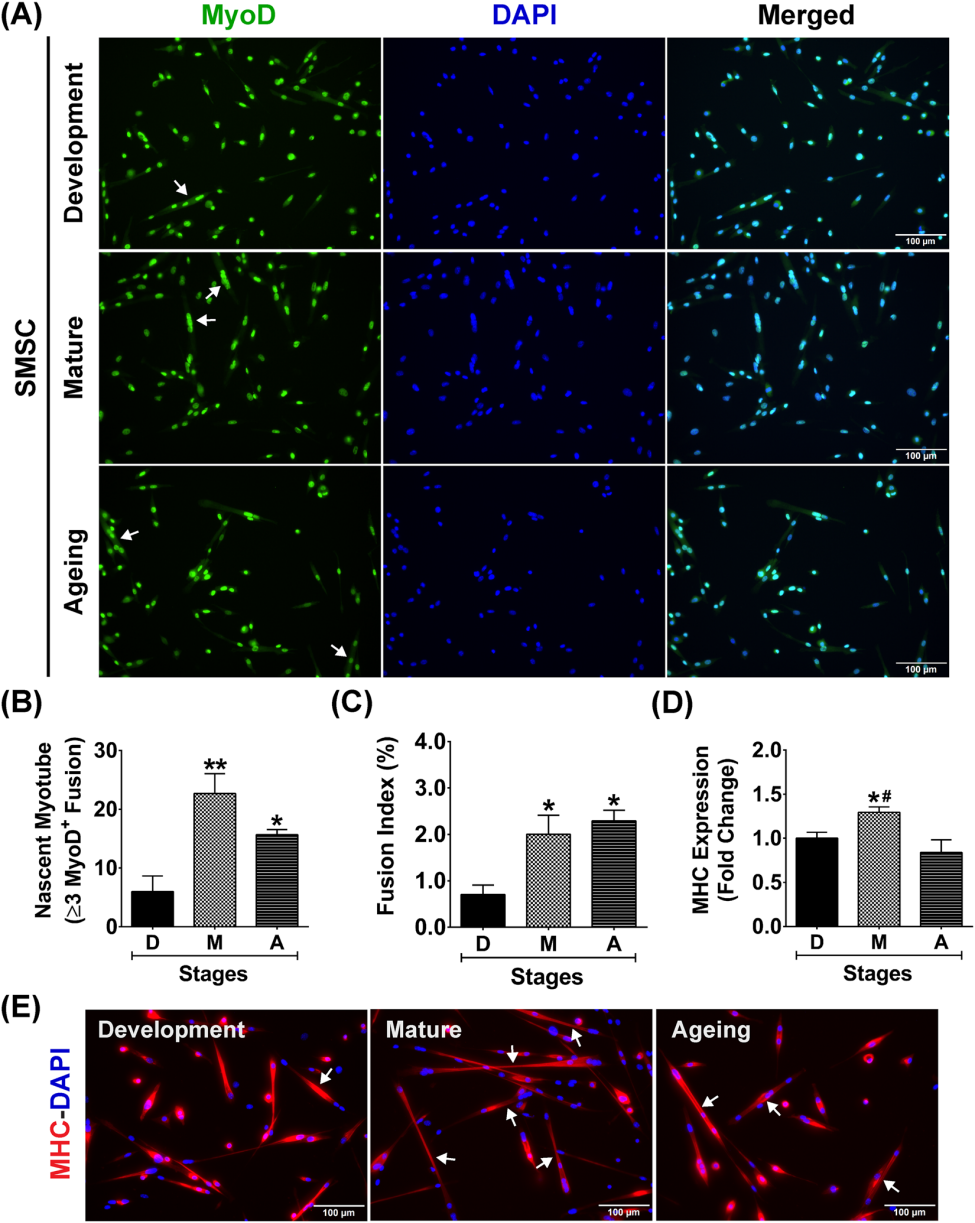
Differentiation characteristics of developmental, mature, and aged SMSCs. (**A**) Representative images of developmental, mature, and aged SMSCs (MyoD^+^ cells) under growth-stimulating conditions for 48 h. Arrows in (**A**) indicate fusion of ≥3 MyoD^+^ nuclei (nascent myotube formation). (**B**–**D**) Quantitative analyses of (**B**) number of nascent myotubes, (**C**) fusion index, and **(D)** MHC protein expression from developmental, mature, and aged SMSCs (n = 3 SMSCs isolated from different mice/growth stage). (**E**) Representative images of MHC staining in developmental, mature, and aged SMSCs under growth-stimulating conditions for 48 h. Arrows in (**E**) indicate nascent myotubes (MHC^+^) that contains ≥3 nuclei fusion. *p < 0.05 and **p < 0.01 compared to developmental stage and ^#^*p* < 0.01 compared to ageing stage. Images were taken at ×200 magnification.

Nuclear VDR protein was non-uniformly expressed in SMSCs (MyoD^+^ cells) under growth-stimulating conditions (Fig. 9B), suggesting that the expression of VDR protein in response to 1α,25(OH)_2_D_3_ treatment could be state-specific. To support this hypothesis, SMSCs strongly expressed VDR protein specifically in the undifferentiated state (Myogenin^−^ cells) as illustrated in Fig. 9C (arrows). This result supports the notion that VDR protein expression is down-regulated in SMSCs undergoing differentiation (Myogenin^+^ cells). Based on this finding, the differentiation characteristics of developmental, mature, and aged SMSCs were investigated to evaluate the diminished response of SMSC under 1α,25(OH)_2_D_3_ treatment during advanced age. The characteristics of SMSCs (MyoD^+^ cells) derived from the different growth stages under growth-stimulating conditions are illustrated in Fig. 10A. Results reveal that developmental SMSC had a significantly lower number of nascent myotubes (≥3 MyoD^+^ nuclei fusion) (Fig. 10B) and fusion index (number of ≥3 MyoD^+^ nuclei fusion/total MyoD^+^ nuclei) (Fig. 10C) than mature and aged SMSCs. These results suggest that developmental SMSC can retain an undifferentiated state under growth-stimulating conditions which corresponded with a substantial increase in VDR protein expression after 1α,25(OH)_2_D_3_ treatment. In contrast, mature and aged SMSCs had a significantly greater number of nascent myotubes (*p* < 0.01 and *p* < 0.05) (Fig. 10B) and a fusion index (*p* < 0.05) (Fig. 10C) compared to developmental SMSC which was associated with a diminished response to 1α,25(OH)_2_D_3_. However, the rapid commitment of SMSC to differentiation was reduced in aged SMSC compared to mature SMSC (*p* < 0.05) (Fig. 10D) as illustrated by MHC protein expression (Fig. 10E). This result was supported by a lower number of aged SMSC compared to mature SMSC (decreased to 58.0 ± 11.2%) in response to growth-stimulating conditions (*p* < 0.05). Altogether, these findings suggest that state-specific VDR protein expression in SMSC could be a primary factor that influencing vitamin D_3_ action in skeletal muscle during advanced age.

## Discussion

This study provides insight about the vitamin D system in skeletal muscle at a tissue and cellular level during advanced age. The primary findings of this work are 1) VDR protein was barely detected in developmental muscle but substantially increased in aged muscle which was more pronounced in fast-glycolytic compared to slow-oxidative muscles; 2) levels of vitamin D-metabolising enzymes (CYP27B1 and CYP24A1) in skeletal muscle were not affected by local VDR protein expression or circulating 25(OH)D_3_ level; and 3) SMSC expressed vitamin D system-related proteins, however, there was a diminished response of SMSC to 1α,25(OH)_2_D_3_ during advanced age due to a rapid commitment of SMSC towards differentiation under growth-stimulating conditions.

Currently, the non-calcemic actions of vitamin D on skeletal muscle have been reported, i.e., development, strength, ageing, and repair^36^. In addition, there is supportive evidence in the beneficial effects of vitamin D on elderly population where muscle mass and function is compromised^20,30^. However, how vitamin D exerts its action and regulates the vitamin D system in skeletal muscle remains unknown. Previous studies have demonstrated undetectable levels of VDR protein in skeletal muscle tissue^37,38^. Subsequent investigations reported that VDR protein expression was detected in normal muscle^10,12,18,21^ and substantially increased during skeletal muscle regeneration^4,6^. The latter findings suggest that skeletal muscle plasticity is associated with SMSC function during repairing process, which could be a primary factor regulating VDR protein expression in skeletal muscle. However, whether vitamin D system-related proteins are differentially regulated in slow-oxidative and fast-glycolytic muscles currently unknown.

In this study, slow-oxidative muscle (soleus) expressed VDR protein at low level during development, which progressively increased during advanced age. In contrast, fast-glycolytic muscle (plantaris) had barely detectable levels of VDR protein expression compared to soleus muscle. However, we observed significant increases in VDR protein expression in plantaris muscle during transition from maturation to ageing. These findings on differential expression of VDR protein between soleus and plantaris muscles during ageing suggest an intrinsic factor related to fibre type composition could contribute to the regulation of VDR protein expression. To support this notion, soleus muscle contains a majority of type I muscle fibres that are associated with a higher number of SMSCs than plantaris muscle^39,40^. SMSCs are a potential source of VDR in skeletal muscle tissue according to a previous investigation^4^. Therefore, the differences in SMSC content during postnatal development could be responsible for the higher level of VDR protein expression in soleus muscle. However, an increase in SMSC activity in plantaris muscle to compensate for type II muscle fibre atrophy during ageing could lead to a substantial increase in VDR protein expression. Since SMSC are required to maintain the myonuclear domain (ratio of cytoplasmic volume to myonuclear number) in atrophic muscle^41^. Additionally, a significant reduction of downstream mTOR signalling protein expression (decreased phosphorylations of 4E-BP1 and p70 S6K1 proteins) was more pronounced in plantaris compared to soleus muscle during ageing supporting a decreased in protein synthesis that requires increased SMSC activity. Altogether, increased SMSC activity could augment VDR protein expression in plantaris compared to soleus muscle during ageing.

Even though our results provide compelling evidence about the up-regulation of VDR protein expression in aged muscle, SMSC as a potential source of VDR expression in skeletal muscle, declined after birth (P0) and underwent quiescence during postnatal development (P21)^32^. Reductions in SMSC content and activity could lead to decreased VDR protein expression in skeletal muscle during postnatal development compared to neonatal stage as previously reported^10^. Intriguingly, the increased VDR protein expression observed in aged muscle compared to developmental muscle is paradoxical. To explain the current findings, an increase in VDR protein expression in aged muscle was associated with increased centronucleated muscle fibres, suggesting an increased number of activated SMSC. SMSC activation in aged muscle might recapitulate SMSC activity during neonatal stages would result in substantially increased VDR protein expression.

To prevent age-related concomitant diseases that could affect an interpretation of the results on vitamin D system in skeletal muscle, the age of mice in this study represents an early stage of ageing without signs of sarcopenia (age-associated loss of muscle mass and function). Therefore, the potential reduction of VDR protein expression during sarcopenia that occurs in very old age should not be excluded. Lower muscle VDR protein expression in sarcopenic patients with a significant reduction of lean body mass and grip strength has been reported^21^. Additionally, a reduced SMSC pool in sarcopenic muscle^42^ could be a potential contributing factor to decreased VDR protein expression during sarcopenia.

In the current study, vitamin D deficiency was detected at development as indicated by serum 25(OH)D_3_ level <20 ng/ml. The low levels of serum 25(OH)D_3_ were associated with low VDR protein expression in developmental muscle and vitamin D-sensitive tissues (kidney and intestine). The temporal expression of VDR protein in skeletal muscle that orchestrates with vitamin D-sensitive tissues suggests an influence of circulating 25(OH)D_3_ level on systemic VDR protein expression during development. In contrast, total serum Ca^2+^ level at development was normal suggesting that low levels of 25(OH)D_3_ and VDR protein expression are sufficient to maintain Ca^2+^ homeostasis at this stage of postnatal development. These results were supported by a previous investigation demonstrating that serum Ca^2+^ remained normal in severe vitamin D deficient patients if serum 25(OH)D concentration not fall below ∼10 nM^43^. Although serum 25(OH)D_3_ was increased toward normal level (∼30 ng/ml) during maturation and maintained during ageing, VDR protein expression in aged muscle was increased independent of serum 25(OH)D_3_ level. These results suggest that the contribution of intrinsic factors (i.e., SMSC activity), in addition to systemic 25(OH)D_3_ level, regulate VDR protein expression in skeletal muscle during ageing.

In contrast to the increased VDR protein expression during advanced age, no significant change in vitamin D-metabolising enzymes (CYP27B1 and CYP24A1) were evident in skeletal muscle at any age. In general, vitamin D-metabolising enzymes in the kidney regulate 1α,25(OH)_2_D_3_ concentration under influence of parathyroid hormone, fibroblast growth factor 23 (FGF23), and circulating 1α,25(OH)_2_D_3_ level^44^. However, changes in serum 25(OH)D_3_ level from development to maturation that could affect systemic 1α,25(OH)_2_D_3_ concentration did not alter CYP27B1 and CYP24A1 protein expression in skeletal muscle. These findings suggest that vitamin D-metabolising enzymes in skeletal muscle might be responsible for the local regulation of vitamin D metabolism but not regulate systemic production of 1α,25(OH)_2_D_3_ or its inactive metabolite (calcitroic acid). Moreover, no associations between VDR and vitamin D-metabolising enzyme expression levels in aged muscle were evident. These results differ from previous investigations showing VDR and CYP24A1 mRNA/protein expression in skeletal muscle cells/tissue are concurrently increased in response to 1α,25(OH)_2_D_3_ treatment, i.e., studies in primary muscle cell culture^10,12^, skeletal muscle cell line^8,45,46^, and regenerating muscle^4^. From this point of view, an increase in VDR protein expression in aged muscle could be due to local factors in skeletal muscle rather than changes in systemic 1α,25(OH)_2_D_3_.

To support the local vitamin D system in skeletal muscle, SMSCs isolated from developmental, mature and aged muscles were shown to express vitamin D system-related proteins (VDR, CYP27B1, and CYP24A1). These findings reveal a potential impact of vitamin D_3_ on SMSC, which are major contributors to skeletal muscle plasticity. Up-regulation of VDR protein expression in SMSC (MyoD^+^ cell) after 1α,25(OH)_2_D_3_ treatment was consistent with previous reports on vitamin D_3_ action in rodent and human primary skeletal muscle cells^10,11,13^. Additionally, cultured SMSC expressed nuclear VDR protein that co-localised with CYP24A1 protein after 1α,25(OH)_2_D_3_ treatment. This result supports previous work regarding regulation of vitamin D_3_ action in SMSC during regeneration through VDR and CYP24A1 activation after intramuscular administration of 1α,25(OH)_2_D_3_^4^. However, the decreased response of SMSC to 1α,25(OH)_2_D_3_ treatment was apparent during advanced age in this study. Alteration of SMSC characteristics during advanced age could influence the effect of vitamin D_3_ on the regulation of VDR protein expression. To support this notion, the rapid commitment to differentiation of mature and aged SMSCs under growth-stimulating conditions might diminish the response to 1α,25(OH)_2_D_3_, as VDR protein is highly expressed in undifferentiated SMSCs (Myogenin^-^) after 1α,25(OH)_2_D_3_ treatment. This novel finding suggests that state-specific regulation of VDR protein expression in SMSC could be a crucial factor affecting vitamin D_3_ action on skeletal muscle during advanced age. To substantiate this finding *in vivo*, conditional knockout models using Cre-Lox recombination targeting myogenic regulatory factors (MRF4, Myf5, MyoD, and myogenin) would be a useful tool to study the state-specific response of SMSC to vitamin D_3_ supplementation at different stages of skeletal muscle growth.

In conclusion, the present study provides insight into the local vitamin D system in skeletal muscle at different stages of growth. We observed significant elevations in VDR protein expression in skeletal muscle during advanced age. This difference was more pronounced in fast-glycolytic compared to slow-oxidative muscle during the transition from maturation to ageing. The substantial increase in VDR protein expression in aged muscle could be impacted by an increase in SMSC activity. Additionally, SMSC express vitamin D system-related proteins to support the action of vitamin D_3_ on the regulation of skeletal muscle plasticity via SMSC. However, the diminished response of SMSC to 1α,25(OH)_2_D_3_ treatment during advanced age could due to the rapid commitment of SMSC towards differentiation under growth-stimulating conditions. In addition, we observed no association of vitamin D-metabolising enzyme (CYP27B1 and CYP24A1) protein expression in skeletal muscle with circulating 25(OH)D_3_ suggesting that skeletal muscle is not the tissue responsible for regulation of systemic 1α,25(OH)_2_D_3_. Taken together, our evidence on vitamin D system in skeletal muscle supports a role for non-calcemic action of vitamin D in this non-classical vitamin D target tissue.

## Materials and Methods

### Animals

Male C57BL/6 mice were obtained from Nomura Siam International Co, Ltd. (Bangkok, Thailand) and assigned into three groups: development (aged 4-week-old), maturation (aged 24- to 25-week-old), and ageing (aged 72- to 76.5-week-old). The maximum age of mice in each growth stage corresponded approximately to 1-month-old, 6-month-old, and 18-month-old study periods, respectively. In each group, mice were allocated for an investigation of vitamin D system in skeletal muscle tissue (*in vivo* study) and SMSC culture (*in vitro* study). Mice were housed in temperature- and humidity-controlled room with a 12:12 light-dark cycle. In addition, mice were fed with CLEA Rodent Diet CE-2 containing 205 IU/100 g vitamin D_3_ (Nomura Siam International Co, Ltd.) and reverse osmosis water ad libitum throughout the study.

### Tissue sample collection

Before tissue sample collection, mouse body weight (g) was recorded to determine body weight at different stages of growth. Soleus and plantaris muscles were dissected and muscle wet weight (mg) was measured using a digital weight scale (Model MS204) (Mettler Toledo, Greifensee, Switzerland). For Western blot and histological/immunohistochemical analyses, muscle samples were frozen in liquid nitrogen or covered with Tissue-Tek O.C.T. compound (4583) (Sakura Finetek, CA, USA) before being frozen in 2-methylbutane (M32631) (Sigma-Aldrich, MO, USA) pre-cooled with liquid nitrogen. In addition, kidney and intestine (duodenum) were collected and frozen in liquid nitrogen to serve as the positive controls for vitamin D system-related protein expression analysis.

### Analyses of serum 25(OH)D_3_/D_2_, 3-Epi-25(OH)D_3_/D_2_, and total Ca^2+^ levels

Serum was obtained from blood placed at room temperature for 30 min followed by centrifugation at 4 °C using refrigerated centrifuge Model 5430 R (Eppendorf, Germany) (3,000 rpm for 15 min). Serum levels of 25(OH)D_3_/D_2_ and 3-Epi-25(OH)D_3_/D_2_ were determined by LC-MS/MS using the MassChrom^®^ 25-OH-Vitamin D_3_/D_2_ in Serum/Plasma kit (Chromsystems Instruments & Chemicals GmbH, Germany). Serum concentration of total Ca^2+^ was analysed using the *o*-cresolphthalein complexone method.

### Histological analysis

Muscle sections at 10 µm thickness were obtained using a cryostat (model CM1850, Leica, Wetzlar, Germany). The sections were stained with Hematoxylin solution modified acc. to Gill II (105175) and Eosin Y (C.I. 45380) (115935) (Merck Millipore, MA, USA) to evaluate the histological structure of muscle fibres, fibre cross-sectional area (CSA), and fibre number. Images were taken at ×100 and ×400 magnifications using an Olympus microscope (BX53) (Olympus, Tokyo, Japan) equipped with a digital camera (DP73) (Olympus, Tokyo, Japan). Fibre CSA and the distribution of fibre size were analysed from 200–300 fibres/individual muscle section. Fibre number was counted from the entire muscle cross-section. The quantitative analyses of fibre CSA and fibre number were performed using ImageJ (National Institutes of Health, Bethesda, MD) and cellSens Dimension software (Olympus, Tokyo, Japan), respectively.

### Immunohistochemistry

Immunostaining protocols were performed following previously described^4^. Briefly, the sections were fixed with 4% paraformaldehyde (PFA) (15713) (Electron Microscopy Sciences, PA, USA), permeabilized with 0.5% Triton X-100 (X100) (Sigma-Aldrich, MO, USA), blocked with mouse IgG blocking reagent (MKB-2213) (Vector Labs, CA, USA), and with 10% normal goat serum (PCN5000) (Invitrogen, CA, USA). Primary and secondary antibodies were used as follows: mouse monoclonal anti-VDR (D-6) (1:100, sc-13133), rabbit polyclonal anti-CYP24A1 (1:100, sc-66851), and rabbit polyclonal anti-CYP27B1 (1:100, sc-67261) (Santa Cruz Biotechnology, CA, USA); rabbit polyclonal anti-fast MHC (1:1,000, ab91506) and mouse monoclonal anti-slow MHC (1:2,000, ab11083) (Abcam, Cambridge, UK); rabbit polyclonal anti-laminin antibody (1:400, L9393) (Sigma-Aldrich, MO, USA); mouse monoclonal anti-ATP synthase (complex V) subunit α (OxPhos) (1:200, 459240), goat anti-mouse Alexa Fluor^®^ 568 IgG (H + L) (1:500, A-11004), and goat anti-rabbit Alexa Fluor^®^ 488 IgG (H + L) (1:500, A-11008) secondary antibodies (Invitrogen, CA, USA). Nuclear localisation was visualized using 4’,6-diamidino-2-phenylindole, dihydrochloride (DAPI) (D1306) (Thermo Fisher Scientific, MA, USA). Stained sections were mounted with antifade mounting medium (H-1000) (Vector Labs, CA, USA). Images were taken using an Olympus microscope (BX53) (Olympus) equipped with a digital camera (DP73) (Olympus) using image acquisition software (cellSens Dimension Desktop, Olympus, Japan) at ×100, ×200, and ×400 magnifications.

### SMSC isolation and culture

An isolation protocol was performed according to a previously described method^47^. Isolated SMSCs were obtained from developmental, mature, and aged muscles and seeded on 2% gelatin-coated T25 flasks and cultured using DMEM supplemented with 20% fetal bovine serum (FBS) (10270-106) (Gibco, NY, USA) and 5 ng/ml of basic fibroblast growth factor (bFGF), human recombinant (GF003) (Merck Millipore, MA, USA) at 37 °C and 5% CO_2_. The pre-plating technique was performed during passaging to eliminate contaminating fibroblasts during the isolation process. The purity of SMSCs was determined by myoblast determination protein (MyoD) expression. Ten randomized fields of cultured SMSCs from developmental, mature, and ageing stages (n = 3 SMSCs isolated from different mice/growth stage) were analysed to evaluate the percentage of MyoD^+^ nuclei [(number of MyoD^+^ nuclei/total nuclei) × 100]. SMSCs at passage 5–6 with >98% of MyoD^+^ nuclei were used in this study.

### Assessment of vitamin D system in SMSC

Developmental, mature, and aged SMSCs were seeded on 2% gelatin-coated 12-well plate and cultured with DMEM + 20% FBS + 5 ng/ml bFGF for 48 h. On the day of treatment, culture media was washed twice with sterile phosphate-buffered saline (PBS) (10010023) (Gibco, NY, USA) and replaced with DMEM + 20% FBS + 10% horse serum (HS) (16050-130) (Gibco, NY, USA) to represent growth-stimulating conditions. SMSCs were treated daily with vehicle (0.1% ethanol) or 1α,25(OH)_2_D_3_ (100 nM) (final concentrations) (71820) (Cayman Chemical, MI, USA) to assess the expression of vitamin D system-related proteins (VDR, CYP27B1, and CYP24A1) under growth-stimulating conditions for 24 h and 48 h, respectively.

### Assessment of SMSC differentiation

Developmental, mature, and aged SMSCs were seeded on 2% gelatin-coated 12-well plate and cultured with DMEM + 20% FBS + 5 ng/ml bFGF for 48 h. After twice washing with PBS, SMSCs were cultured in growth-stimulating conditions (DMEM + 20% FBS + 10% HS) for 48 h. Thereafter, MyoD and myogenin protein expression was determined to investigate the differentiation characteristics of SMSCs under growth-stimulating conditions. Quantitative analysis was performed using image acquisition software (cellSens Dimension Desktop, Olympus, Japan). MyoD-DAPI stained images (10 randomized images at ×200 magnification) were used to analyse the nascent myotube formation (≥3 MyoD^+^ nuclei fusion) and fusion index (number of ≥3 MyoD^+^ nuclei fusion/total MyoD^+^ nuclei). Quantification of MHC protein expression and cell number were analysed using MHC-DAPI stained images (15 randomized images at ×100 magnification).

### Immunocytochemistry

Cells were fixed with 4% PFA, permeabilized with 0.1% Triton X-100, and blocked for non-specific staining with 5% normal goat serum. The primary and secondary antibodies were used as follows: mouse monoclonal anti-MyoD (G-1) antibody (1:500, sc-377460), mouse monoclonal anti-myogenin (F5D) antibody (1:200, sc-12732), mouse monoclonal anti-VDR (D-6) (1:200, sc-13133), rabbit polyclonal anti-CYP24A1 (1:500, sc-66851), and rabbit polyclonal anti-CYP27B1 (1:500, sc-67261) (Santa Cruz Biotechnology, CA, USA); mouse monoclonal anti-myosin heavy chain antibody (MHC) (1:500, 05-716) (Upstate, CA, USA); goat anti-mouse Alexa Fluor^®^ 488 IgG1 (1:500, A-21121), goat anti-mouse Alexa Fluor^®^ 568 IgG2a (1:500, A-21134), goat anti-mouse Alexa Fluor^®^ 488 IgG2b (1:500, A-21141), goat anti-mouse Alexa Fluor^®^ 568 IgG (H + L) (1:500, A-11004), and goat anti-rabbit Alexa Fluor^®^ 488 IgG (H + L) (1:500, A-11008) (Invitrogen, CA, USA). Nuclei were stained with DAPI to delineate nuclear localisation. Representative images were taken at ×200 and ×400 magnifications using an Olympus Inverted Fluorescence Microscope Model IX83 (Olympus, Tokyo, Japan) equipped with ORCA-Flash 2.8 Digital CMOS Camera (C11440) (Hamamatsu Photonics, Hamamatsu, Japan).

### Western blot analysis

Proteins from tissue samples (skeletal muscle, kidney, and intestine) and SMSC samples were extracted using RIPA buffer (50 mM Tris pH 7.5, 150 mM NaCl, 1 mM EDTA, and 1% Triton^®^X-100) supplemented with protease inhibitor (1:100) (P8340) (Sigma-Aldrich, MO, USA) and phosphatase inhibitor cocktails (1:100) (524625) (Merck Millipore, MA, USA). Protein concentrations were determined using BCA assay and the optical density was measured using Spark™ 10 M multimode microplate reader (Tecan Trading AG, Männedorf, Switzerland). After denaturing the protein samples by heating at 60 °C for 10 min, 30 µg (tissue samples) or 20 µg (SMSC samples) of protein was loaded into SDS-polyacrylamide gel (4% stacking and 10% separating). Protein samples were transferred to PVDF membrane, blocked with 5% non-fat milk, and probed with primary antibodies as follows: mouse monoclonal anti-VDR (D-6) (1:200, sc-13133), rabbit polyclonal anti-CYP24A1 (1:200, sc-66851), and rabbit polyclonal anti-CYP27B1 (1:200, sc-67261) (Santa Cruz Biotechnology, CA, USA); rabbit polyclonal anti-glyceraldehyde-3-phosphate dehydrogenase (GAPDH) (1:5,000, ABS16) (Merck Millipore, MA, USA); rabbit polyclonal anti-phospho-4E-BP1 Ser65 (1:1,000, 9451), rabbit monoclonal anti-4E-BP1 (1:1,000, 9644), mouse monoclonal anti-phospho-p70 S6K1 Thr389 (1:1,000, 9206), and rabbit monoclonal anti-p70 S6K1 (1:1,000, 2708) (Cell Signaling, MA, USA). Membranes were incubated with goat anti-rabbit peroxidase-conjugated antibody (1:7,000, AP132P) and goat anti-mouse peroxidase-conjugated antibody (1:7,000, AP124P) (Merck Millipore, MA, USA). Protein bands were visualized using chemiluminescence horseradish peroxidase detection reagent (WBLUR0100) (Merck Millipore, MA, USA) and exposed to UltraCruz Autoradiography Film (sc-201696) (Santa Cruz Biotechnology, CA, USA). Quantification of the protein expression levels was performed using Image J (National Institutes of Health, Bethesda, MD).

### Statistical analysis

Data are expressed as means and standard errors of the mean (means ± SEM). Significant differences among groups were determined using One-way ANOVA with Tukey’s post-hoc test, independent-samples Kruskal-Wallis test, or independent sample *t*-test. Correlation analysis was evaluated using Pearson correlation coefficient. All statistical tests were performed using SPSS and *p* < 0.05 represents the significant difference between groups.

### Ethical approval and informed consent

Experimental procedures in animals were performed in accordance with institutional guidelines for the care and use of laboratory animals. The animal protocol has been approved by the Animal Care and Use Committee of Faculty of Science, Mahidol University (SCMU-ACUC; Protocol ID MUSC59-008-341).

## Supplementary information

Supplementary Information.

## Data Availability

The datasets generated during and/or analysed during the current study are available from the corresponding author on reasonable request.
