# Supplementary material for: Regulation of vitamin D system in skeletal muscle and resident myogenic stem cell during development, maturation, and ageing
Source: Sci Rep. 2020 May 19;10:8239. doi: 10.1038/s41598-020-65067-0 (PMC7237670; doi:10.1038/s41598-020-65067-0)
Supplement: Supplementary file 1 — Supplementary Information. [file 41598_2020_65067_MOESM1_ESM.pdf]

Supplementary Information (Manuscript SREP-19-40452A)

Regulation of vitamin D system in skeletal muscle and resident myogenic stem cell during development, maturation, and ageing

Ratchakrit Srikuea[1*], Muthita Hirunsai[2], Narattaphol Charoenphandhu[1,3,4,5]

[1]Department of Physiology, Faculty of Science, Mahidol University, Bangkok 10400, Thailand

[2]Department of Biopharmacy, Faculty of Pharmacy, Srinakharinwirot University, Nakhon Nayok 26120, Thailand

[3]Center of Calcium and Bone Research (COCAB), Faculty of Science, Mahidol University, Bangkok 10400, Thailand

[4]Institute of Molecular Biosciences, Mahidol University, Nakhon Pathom 73170, Thailand

[5]The Academy of Science, The Royal Society of Thailand, Dusit, Bangkok 10300, Thailand

Address for correspondence:

Ratchakrit Srikuea, Ph.D.

Department of Physiology, Faculty of Science

Mahidol University, Bangkok 10400, Thailand

Tel: +66 22015518

Fax: +66 23547154

E-mail: ratchakrit.sri@mahidol.ac.th

Supplementary Figure S1. Original data representing VDR and GAPDH protein expression in Figure 4A-D. (Srikuea *et al.*)

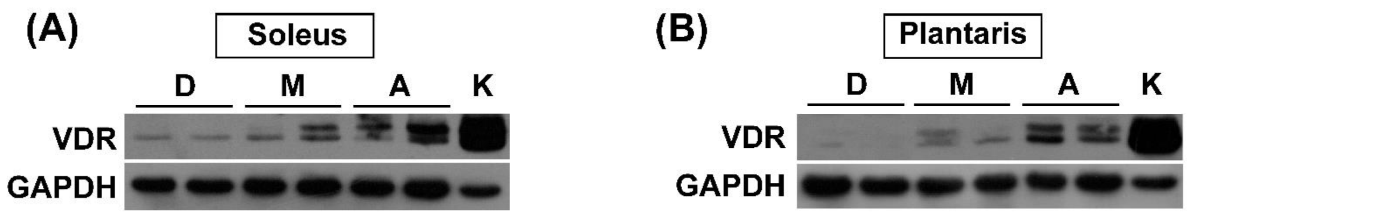

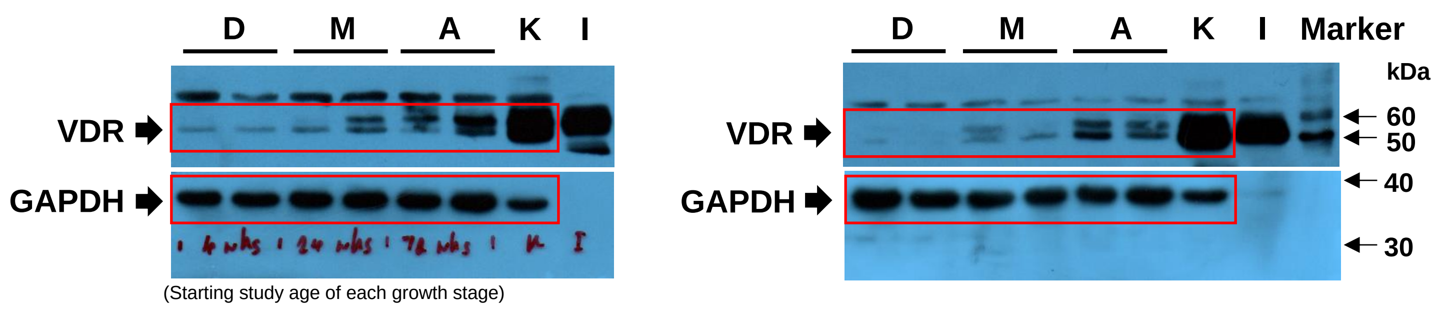

D (Developmental stage), M (Maturation stage), and A (Ageing stage)
K = Kidney and I = Intestine (Positive controls for VDR protein expression)

Note: Membrane obtained from the same gel was cut at ~40 kDa to probe separately with VDR and GAPDH antibodies. GAPDH protein expression was barely detected in intestine samples when the same exposure duration was used to detect GAPDH protein expression in skeletal muscle (soleus and plantaris) and kidney samples.

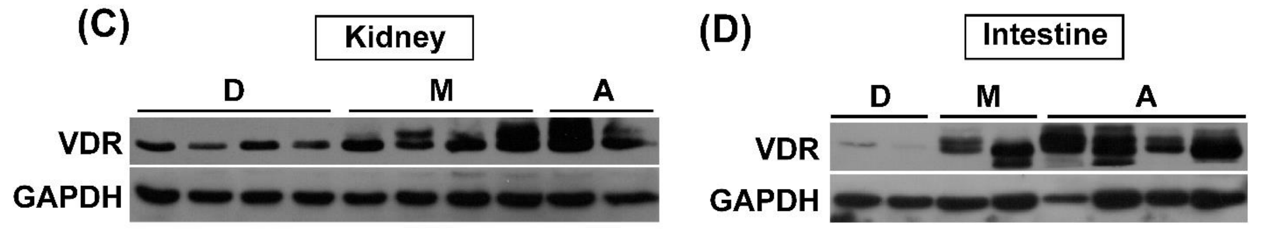

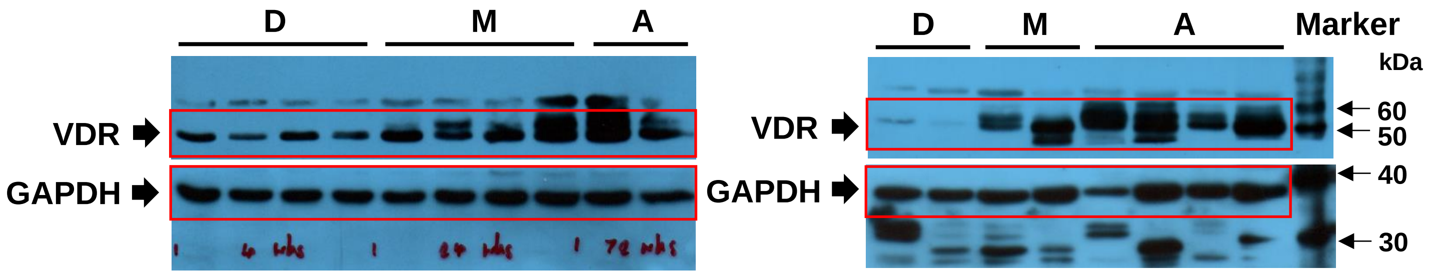

Note: Membrane obtained from the same gel was cut at ~40 kDa to probe separately with VDR and GAPDH antibodies. Prolonged exposure duration was used to detect GAPDH protein expression in intestine samples that cause an induction of non-specific bands.

Supplementary Figure S2. Original data representing CYP27B1, CYP24A1, and GAPDH protein expression in Figure 5A-B. (Srikuea *et al.*)

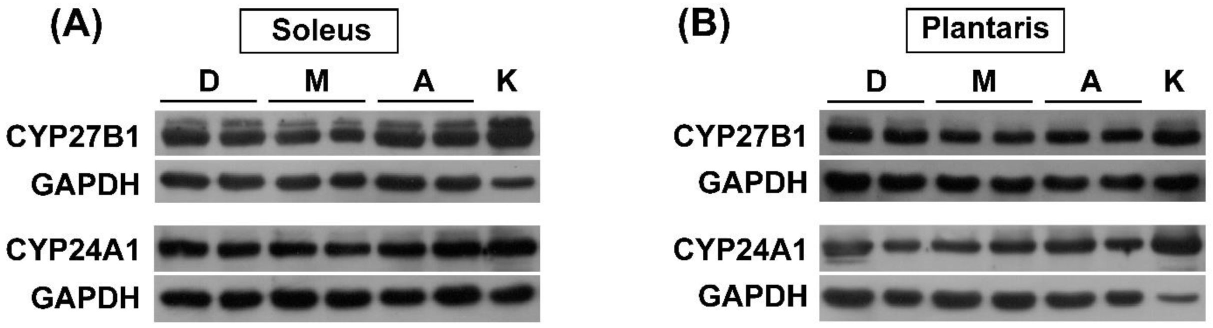

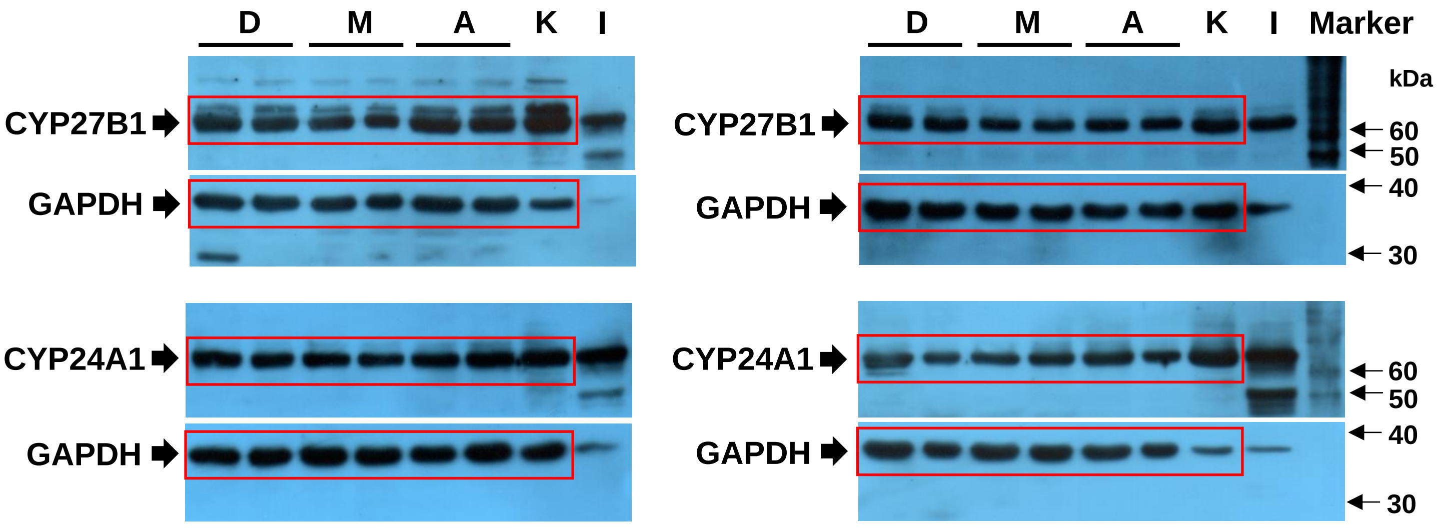

D (Developmental stage), M (Maturation stage), and A (Ageing stage)
K = Kidney (Positive control for CYP27B1 and CYP24A1 protein expression) and I = Intestine

Note: Membrane obtained from the same gel was cut at ~40 kDa to probe separately with CYP27B1/CYP24A1 and GAPDH antibodies. GAPDH protein expression was barely detected in intestine samples when the same exposure duration was used to detect GAPDH protein expression in skeletal muscle (soleus and plantaris) and kidney samples.

Supplementary Figure S3. Original data representing phospho-4E-BP1/4E-BP1 and phospho-p70 S6K1/p70 S6K1 protein expression in Figure 7C-D. (Srikuea *et al.*)

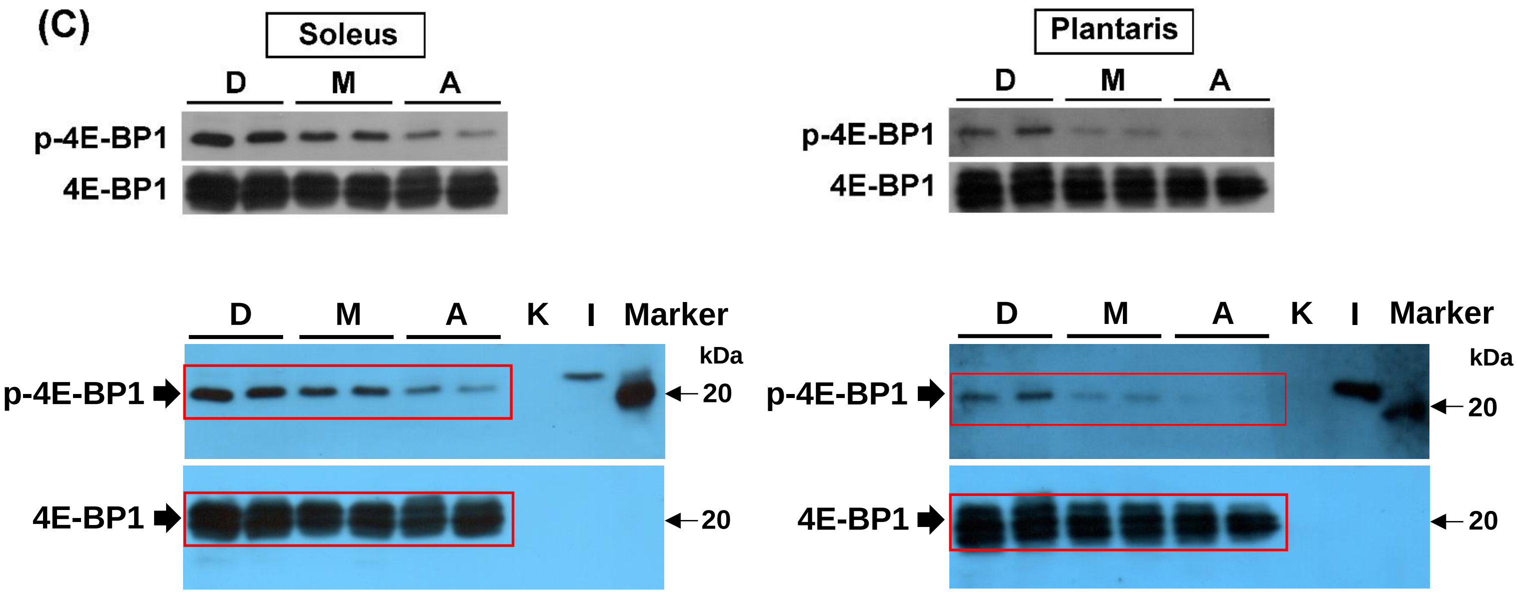

D (Developmental stage), M (Maturation stage), and A (Ageing stage)
K = Kidney and I = Intestine

Note: Membrane was probed with phospho-4E-BP1 antibody and then stripped to probe with 4E-BP1 antibody.

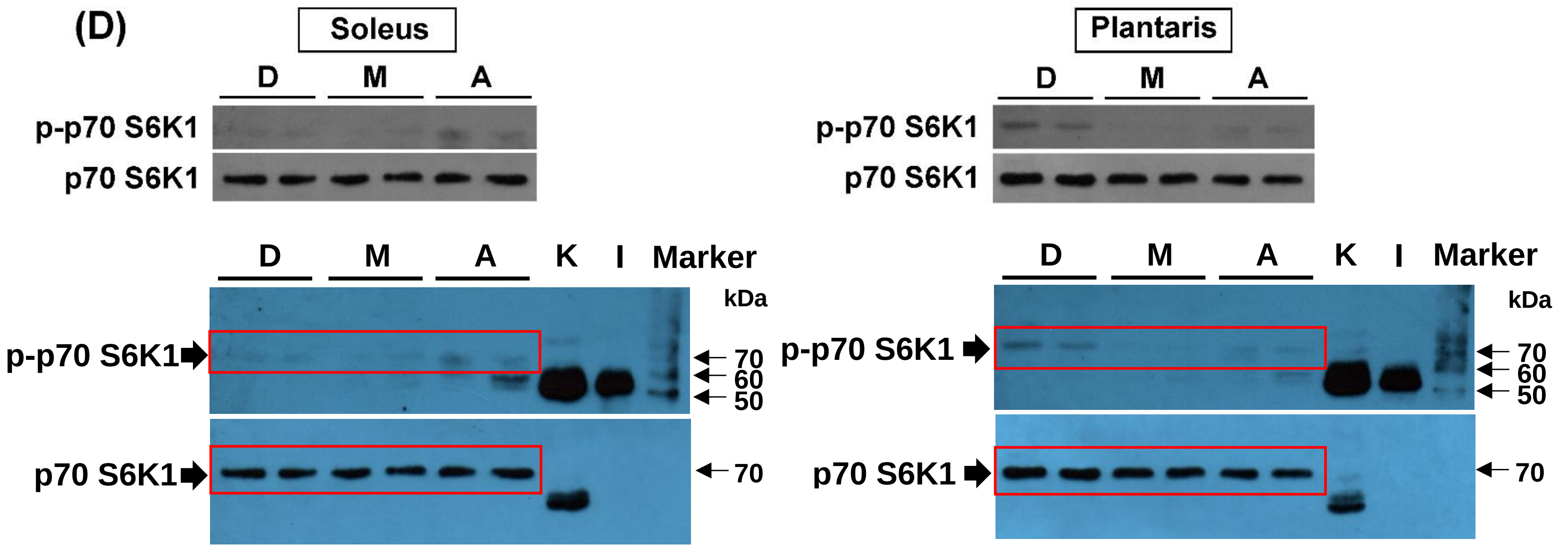

D (Developmental stage), M (Maturation stage), and A (Ageing stage)
K = Kidney and I = Intestine

Note: Membrane was probed with phospho-p70 S6K1 antibody and then stripped to probe with p70 S6K1 antibody.

Supplementary Figure S4. Original data representing VDR and GAPDH protein expression in Figure 9A. (Srikuea *et al.*)

(A)

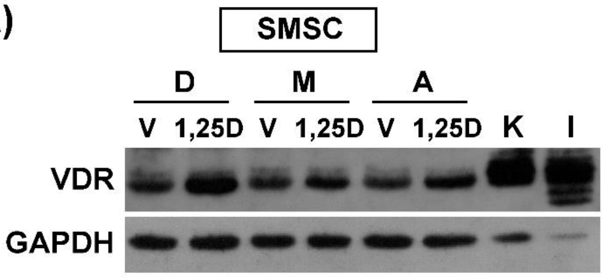

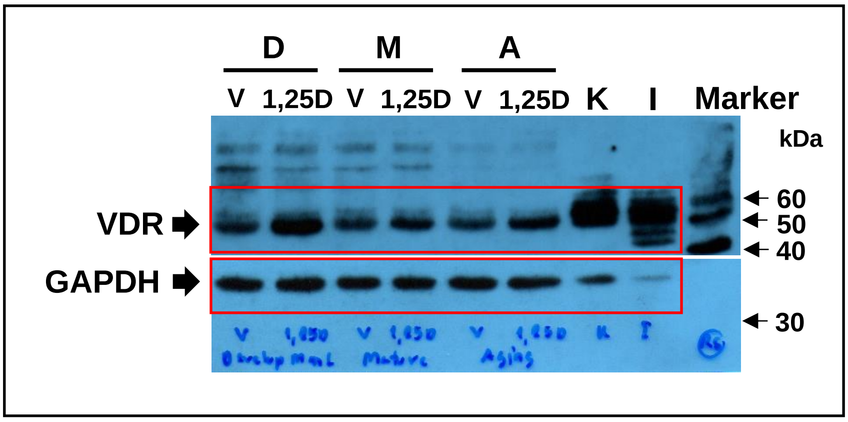

D (Developmental stage), M (Maturation stage), and A (Ageing stage)
K = Kidney and I = Intestine (Positive controls for VDR protein expression)

Note: Membrane obtained from the same gel was cut at ~40 kDa to probe separately with VDR and GAPDH antibodies.
